# Diagnosis of an intraductal papillary neoplasm of the bile duct with fibrovascular stalks using detective flow imaging

**DOI:** 10.1055/a-2134-9350

**Published:** 2023-08-30

**Authors:** Shunsuke Omoto, Mamoru Takenaka, Tomohiro Fukunaga, Ayana Okamoto, Yoriaki Komeda, Seok Jeong, Masatoshi Kudo

**Affiliations:** 1 Department of Gastroenterology and Hepatology, Kindai University Faculty of Medicine, Osaka-Sayama, Japan; 2 Department of Gastroenterology and Hepatology, Yodogawa Christian Hospital, Osaka, Japan; 3 Division of Gastroenterology, Department of Internal Medicine, Inha University Hospital, Inha University School of Medicine, Incheon, South Korea

Intraductal papillary neoplasm of the bile duct (IPNB) is a type of epithelial tumor characterized by papillary proliferation within the bile duct. It is defined histologically as a papillary or villous neoplasm covering the delicate fibrovascular stalks within the bile ducts 1 2 ; however, imaging of the fibrovascular stalks has not previously been reported in IPNB. Recent studies have reported on the usefulness of detective flow imaging (DFI) during endoscopic ultrasound (EUS) for the detailed evaluation of vessels in pancreaticobiliary disease, without the use of contrast agents 3 4 5 . Herein, we describe the usefulness of DFI in identifying the fibrovascular stalks in IPNB.

The patient was a 79-year-old man referred to our hospital for detailed examination of intrahepatic bile duct dilatation. Marked dilatation of the left intrahepatic bile duct and a suspected intrahepatic bile duct tumor were observed on contrast-enhanced computed tomography and magnetic resonance cholangiopancreatography ( Fig. 1 ). EUS confirmed the presence of a tumor with papillary growth within the dilated bile duct. Evaluation of tumor hemodynamics using enhanced flow (e-Flow) imaging failed to detect blood flow signals within the tumor ( Fig. 2 ). Even evaluation with contrast-enhanced harmonic EUS showed that homogeneous enhancement of the tumor made the evaluation of tumor hemodynamics impossible ( Fig. 3 ). In contrast, on DFI, dendritic vessels were observed within the tumor, indicative of a possible fibrovascular stalk ( Fig. 4 ). Surgical resection of the left liver lobe and extrahepatic bile ducts confirmed the presence of fibrovascular stalks in the tumor and therefore the diagnosis of IPNB ( Fig. 5 ; Video 1 ).

**Fig. 1:**
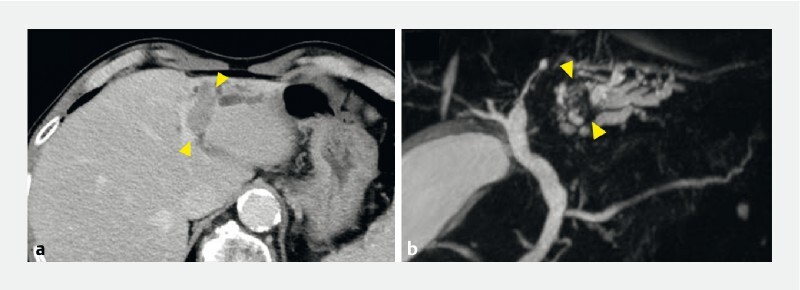
Marked dilatation of the left intrahepatic bile duct and a suspected intrahepatic bile duct tumor (yellow arrowheads) are shown on: **a** contrast-enhanced computed tomography; **b** magnetic resonance cholangiopancreatography.

**Fig. 2:**
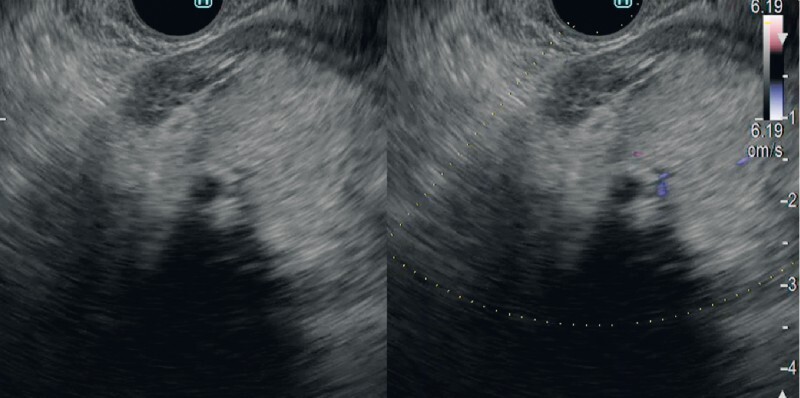
Views during enhanced flow (e-Flow) imaging for evaluation of tumor hemodynamics showing failure to detect blood flow signals within the tumor.

**Fig. 3:**
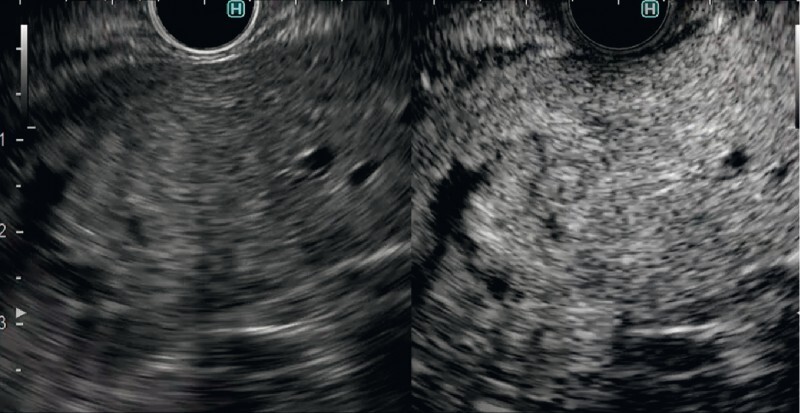
Contrast-enhanced harmonic endoscopic ultrasound image showing homogeneous enhancement of the tumor that makes evaluation of tumor hemodynamics impossible.

**Fig. 4:**
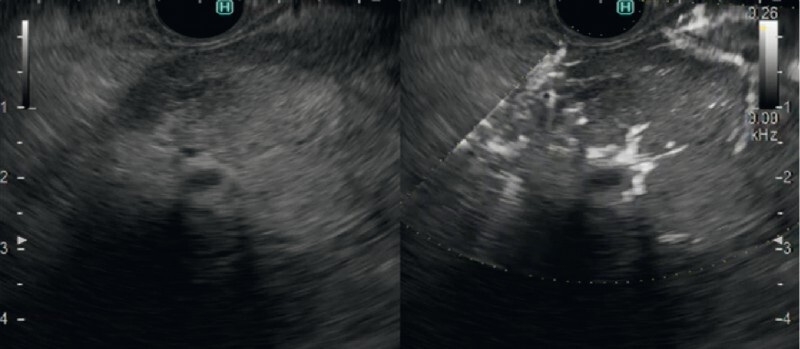
View during detective flow imaging (DFI) showing dendritic vessels within the tumor, which are indicative of a possible fibrovascular stalk.

**Fig. 5:**
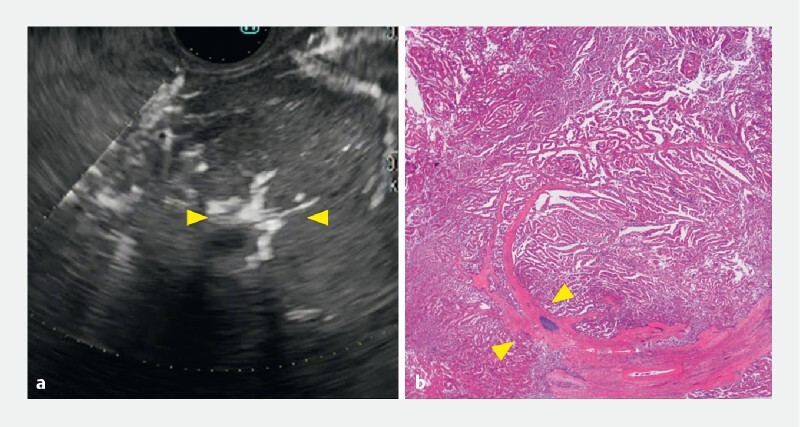
Fibrovascular stalks are seen in the tumor (yellow arrowheads), which was confirmed to be an intraductal papillary neoplasm of the bile duct, on: **a** detective flow imaging; **b** microscopic appearance of the surgically resected left liver lobe and extrahepatic bile ducts.

**Video 1** Detective flow imaging (DFI), a novel imaging technique for low velocity blood flow, is used to identify the fibrovascular stems in intraductal papillary neoplasm of the bile duct (IPNB) that could not be detected by conventional imaging modalities.


To our knowledge, this is the first report on the use of DFI to detect dendritic vessels within an intrahepatic bile duct tumor. DFI, which can capture tumor hemodynamics not detectable by conventional blood flow imaging, may be useful in differentiating intrahepatic bile duct tumors, including IPNB.

Endoscopy_UCTN_Code_TTT_1AS_2AD

## Notes

Endoscopy E-Videos

https://eref.thieme.de/e-videos
*E-Videos* is an open access online section of the journal *Endoscopy* , reporting on interesting cases and new techniques in gastroenterological endoscopy. All papers include a high-quality video and are published with a Creative Commons CC-BY license. Endoscopy E-Videos qualify for HINARI discounts and waivers and eligibility is automatically checked during the submission process. We grant 100% waivers to articles whose corresponding authors are based in Group A countries and 50% waivers to those who are based in Group B countries as classified by Research4Life (see: https://www.research4life.org/access/eligibility/ ). This section has its own submission website at https://mc.manuscriptcentral.com/e-videos
